# Randomized Controlled Trial of Ultrasound-guided Fluid Resuscitation of Sepsis-Induced Hypoperfusion and Septic Shock

**DOI:** 10.5811/westjem.2020.11.48571

**Published:** 2021-02-10

**Authors:** Khrongwong Musikatavorn, Poj Plitawanon, Suthaporn Lumlertgul, Khuansiri Narajeenron, Dhanadol Rojanasarntikul, Tanawat Tarapan, Jutamas Saoraya

**Affiliations:** * Chulalongkorn University and King Chulalongkorn Memorial Hospital, The Thai Red Cross Society, Department of Emergency Medicine, Faculty of Medicine, Bangkok, Thailand; † Chulalongkorn University and King Chulalongkorn Memorial Hospital, Thai Red Cross Society, Department of Medicine, Faculty of Medicine, Bangkok, Thailand; ‡ Chulalongkorn University, Faculty of Medicine, Division of Academic Affairs, Bangkok, Thailand

## Abstract

**Introduction:**

The ultrasound measurement of inferior vena cava (IVC) diameter change during respiratory phase to guide fluid resuscitation in shock patients is widely performed, but the benefit on reducing the mortality of sepsis patients is questionable. The study objective was to evaluate the 30-day mortality rate of patients with sepsis-induced tissue hypoperfusion (SITH) and septic shock (SS) treated with ultrasound-guided fluid management (UGFM) using ultrasonographic change of the IVC diameter during respiration compared with those treated with the usual-care strategy.

**Methods:**

This was a randomized controlled trial conducted in an urban, university-affiliated tertiary-care hospital. Adult patients with SITH/SS were randomized to receive treatment with UGFM using respiratory change of the IVC (UGFM strategy) or with the usual-care strategy during the first six hours after emergency department (ED) arrival. We compared the 30-day mortality rate and other clinical outcomes between the two groups.

**Results:**

A total of 202 patients were enrolled, 101 in each group (UGFM vs usual-care strategy) for intention-to-treat analysis. There was no significant difference in 30-day overall mortality between the two groups (18.8% and 19.8% in the usual-care and UGFM strategy, respectively; *p* > 0.05 by log rank test). Neither was there a difference in six-hour lactate clearance, a change in the sequential organ failure assessment score, or length of hospital stay. However, the cumulative fluid amount given in 24 hours was significantly lower in the UGFM arm.

**Conclusion:**

In our ED setting, the use of respiratory change of IVC diameter determined by point-of-care ultrasound to guide initial fluid resuscitation in SITH/SS ED patients did not improve the 30-day survival probability or other clinical parameters compared to the usual-care strategy. However, the IVC ultrasound-guided resuscitation was associated with less amount of fluid used.

## INTRODUCTION

Sepsis is a significant burden for emergency departments (ED) worldwide.^1^–^3^ Moreover, it has a high mortality rate, especially in those with sepsis-induced tissue hypoperfusion (SITH) and septic shock (SS).^4^–^7^ The initial treatment emphasizes early recognition, prompt administration of antibiotics, and the restoration of hemodynamic status with fluid resuscitation and vasopressor therapy.^8^ Treating patients with SITH/SS with a “usual-care” strategy, which includes prompt administration of isotonic crystalloid at the empirical amount of 30 milliliters per kilogram (mL/kg), has been proven to provide clinical outcomes similar to those of protocol-based therapies in large, well-designed clinical trials.^9^–^11^ However, either excessive fluid bolus or inadequate fluid administration during the initial resuscitation is associated with increased mortality in SS patients.^12^–^16^

Inferior vena cava (IVC) diameter measurement during respirophasic change is widely used to help physicians predict the fluid responsiveness in shock patients and is reasonable to tailor fluid therapy during the resuscitation.^17^–^20^ However, its benefit in reducing the mortality of sepsis patients remains unclear. The primary aim of this study was to evaluate the 30-day mortality outcome of patients with SITH/SS who were treated with ultrasound-assisted fluid management using the respirophasic change of the IVC during the first six hours compared with those who were treated with the “usual-care” strategy. Secondary outcomes were the six-hour lactate clearance, amount of intravenous (IV) fluid, rate of vasopressor and mechanical ventilator (MV) use, and change in sequential organ failure (SOFA) score at 72 hours in the two groups.

## METHODS

### Study Design and Setting

This randomized controlled trial was conducted in a 1,500-bed urban, university-affiliated tertiary-care hospital. This ED has more than 80,000 new patient visits per year. Our institutional review board approved this study, and written informed consent was required for trial participation. Patient recruitment started in January 2017 and concluded at the end of January 2020. This study was registered at ClinicalTrials.gov (identifier NCT03020407).

### Selection of participants

Adult (≥18 years old) nontraumatic SITH/SS patients (see Study definitions) who presented to the ED during the study period were enrolled in the study. Patient eligibility was assessed by emergency physicians during all work shifts. Patients were excluded if they met any of the following criteria: 1) congestive pulmonary edema or known poor systolic cardiac function (left ventricular ejection fraction ≤ 40%); 2) known right heart pathology; 3) had or were suspected of having marked ascites, significant bowel dilatation, or conditions that could cause abdominal hypertension; 4) body mass index ≥ 30 kilograms/meters squared; 5) concomitant attack of a severe airway disease (eg, asthma or chronic obstructive pulmonary disease that may have confounded the IVC interpretation; 6) IVC could not be identified, or its diameter could not be measured correctly; 7) end-stage renal disease with or without dialysis; 8) noninfectious disease as a final diagnosis; 9) pregnancy; 10) were referred from or treated at other healthcare facilities; 11) active hemorrhage; 12) duplicated or multiple case visits; 13) had a living will stating “do not resuscitate”; and 14) declined to consent.

**Population Health Research Capsule**

What do we already know about this issue?

Ultrasonographic inferior vena cava (IVC) diameter measurement is used to tailor fluid therapy in shock patients. Its benefit in reducing sepsis mortality is unclear.

What was the research question?

To compare the 30-day mortality of sepsis patients treated with IVC ultrasound-guided resuscitation and the usual-care strategy.

What was the major finding of the study?

IVC ultrasound-guided resuscitation did not improve survival of sepsis patients but was associated with less fluid volume.

How does this improve population health?

Assessment of ultrasonographic IVC diameter change did not affect overall survival of patients with sepsis.

### Study Definitions

In this study we specifically defined patients with SITH as those with infections and systolic blood pressure equal to or less than 90 millimeters of mercury (mm Hg) or initial lactate equal to or greater than 2 millimoles/liter (mmol/L) at ED presentation. However, we used the most recent definitions of septic shock and other related terms recommended in the literature.^21^ In brief, patients with septic shock are defined as those who require a vasopressor to maintain a mean arterial pressure of 65 mm Hg or greater and whose serum lactate level is greater than 2 mmol/L in the absence of hypovolemia. The venous or arterial lactate level was obtained and followed up using the same method in each individual. Six-hour lactate clearance (%) is calculated as [(initial lactate – lactate at 6 hours)/initial lactate] ×100%. To follow the deterioration or improvement of organ dysfunction of a patient, the sequential organ failure assessment (SOFA) score was determined at ED presentation and at 72 hours after treatment. The SOFA scoring system is described elsewhere. 22–23

### Study Protocol

In our protocol we prepared the preplanned, permuted block-of-four randomization list that was blinded to the investigators before patient allocation. Randomization was set at a 1:1 ratio of the ultrasound-guided and usual-care arms. When an eligible patient was identified and informed consent was obtained, demographic data, preexisting condition, bloodwork, diagnostic investigations, microbiologic workups, and blood lactate were collected at ED arrival (hour 0). Prompt empirical antibiotics were given to every patient within one hour after ED arrival. Then, the patient was rapidly assigned in accordance with the randomization and treated with one of the two treatment strategies as follows:

#### Ultrasound-guided fluid management (UGFM) strategy

In this treatment arm, the treating emergency physician promptly assessed the IVC diameter to obtain the IVC collapsibility index (IVCCI) (or distensibility index, IVCDI; see below for the description, formulation and measurement method) of each patient while venous access was performed and initial laboratory specimens were collected. A previous study showed that IVCCI > 40% was strongly associated with fluid responsiveness.^24^ Accordingly, the patient was given a 10 milliliters (mL)/kg bolus of 0.9% normal saline solution (NSS) without delay if an IVCCI > 40% was discovered, and serial measurements were immediately performed after each IV bolus was achieved an IVCCI < 40% during our protocol. Then, the rate of IV fluid administration was maintained based on the individual’s condition. If the patients in this arm subsequently required endotracheal intubation and MV with sedation within six hours after initiation of therapy, the IVCDI was measured as a replacement for IVCCI. The same amount of NSS was given when IVCDI > 18%^17^ until IVCDI < 18% was achieved. The IVC evaluation was serially performed and recorded every two hours until six hours after ED presentation. The same treatment protocol was repeated when the threshold of IVCCI (or IVCDI) percentage for potential fluid responsiveness was identified.

#### Inferior vena cava diameter measurement and indicators of fluid responsiveness

In our protocol, IVC was identified in longitudinal section in the subcostal area using the curvilinear or phased array transducers (cardiac) of a standard ultrasound machine. The selected area of IVC diameter measurement was set at 2 centimeters distal to the confluence of the hepatic vein by M-mode coupled with two-dimensional mode on frozen screen images using the Sonosite X-porte (Fujifilm Sonosite, Inc., Bothell, WA). All treating physicians including attending staff and residents regularly participated in hands-on training twice a year (as usual basis) by a qualified international instructor in critical care ultrasonography (the third author). The residents who were allowed to perform the study protocol required at least six months exposure in real clinical experience and had passed formal performance evaluation on ultrasonographic IVC measurement. If the patient was breathing spontaneously, the IVCCI, which reflects the decrease in IVC diameter on spontaneous inspiration, was used. IVCCI is calculated as follows:

$$[(\text{IVC }{\text{diameter}}_{\text{max}}-\text{IVC }{\text{diameter}}_{\text{min}})/\text{IVC }{\text{diameter}}_{\text{max}}]\times 100\%.$$

If the patient required MV for respiratory support, the IVCDI, which reflects the increase in IVC diameter on mechanical inspiration, was used. IVCDI is calculated as follows:

$$[(\text{IVC }{\text{diameter}}_{\text{max}}-\text{IVC }{\text{diameter}}_{\text{min}})/\text{IVC }{\text{diameter}}_{\text{min}}]\times 100\%.$$

Sample images of ultrasonographic landmark and respirophasic diameter changes of an IVC during volume expansion are shown in Figures S1A and S1B in the Supplemental material.

#### Usual-care strategy

Patients were promptly treated with 30 mL/kg loading of NSS in this treatment arm. After the

NSS bolus, treatment with either the additional IV fluid or a vasopressor was given at the physicians’ discretion during the six-hour study period. The threshold for the need of a vasopressor was set at mean arterial pressure below 65 mm Hg if a patient did not respond to fluid therapy during each treatment protocol, and the time of vasopressor administration was noted. However, ancillary fluid administration was allowed at treating physicians’ judgment in both treatment arms. Other adjunctive therapies, such as colloid administration, central venous catheterization, or surgical removal of the infectious source, were not prohibited in our protocol and were used at the discretion of the treating physicians. The study patients were closely monitored while we recorded their clinical parameters every two hours for study purposes. Our resuscitative study protocol stopped at six hours after initiation of the treatment. After this period, patients were treated according to the physicians’ judgment.

### Outcome Measurements

At six hours after treatment, the cumulative fluid volume was recorded, and blood lactate was obtained for lactate clearance calculation. At 72 hours after ED presentation, the cumulative fluid volume from the initial presentation was again recorded, and the patients were followed for clinical condition evaluation and blood chemistry tests to calculate the SOFA score and assess its change from the hour-zero baseline. The in-hospital requirement and time to start renal replacement therapy or MV were followed and recorded by searching the electronic data summary of a patient. The indication to initiate these life-saving procedures was at the discretion of the treating physicians. To identify the deceased patients for mortality analysis, we retrieved the electronic database of in- and outpatient clinical records or made a telephone call to the patients or their personal contact in every case at 30 days after the day of hospital presentation. The clinical data retrieval was performed and recorded by the trained non-investigators.

### Data Analysis

#### Sample-size determination

According to the results of large trials of septic shock treatment, the 90-day mortality was 30% in the usual-care group.^9^–^11^ Based on this information, we calculated that a sample of 254 patients would have a power of 80% to detect a relative reduction of 50% in risk (15 percentage points of absolute risk reduction) in the UGFM group, allowing for a loss to follow-up or withdrawal of 5%. The target number for primary outcome analysis would be 121 patients per group. One interim analysis was performed after the enrollment of 50% of the patients, with the use of a two-sided symmetric O’Brien–Fleming (or alpha spending method) design.^25^

#### Statistical analysis

We used Stata version 14.0 (StataCorp LLC, College Station, TX) for all statistical tests and production of graphics. The normally and non-normally distributed data were analyzed using the two independent-samples t test and Mann-Whitney U test, respectively. A χ^2^ test with odds ratio (OR) was performed to compare the proportions between the groups. No data were imputed for any missing information. We used the Kaplan-Meier curve and the log-rank test to compare the 30-day mortality between the treatment arms. All tests were two-sided for superiority testing and considered statistically significant at a *p* < 0.05.

## RESULTS

**Figure 1:**
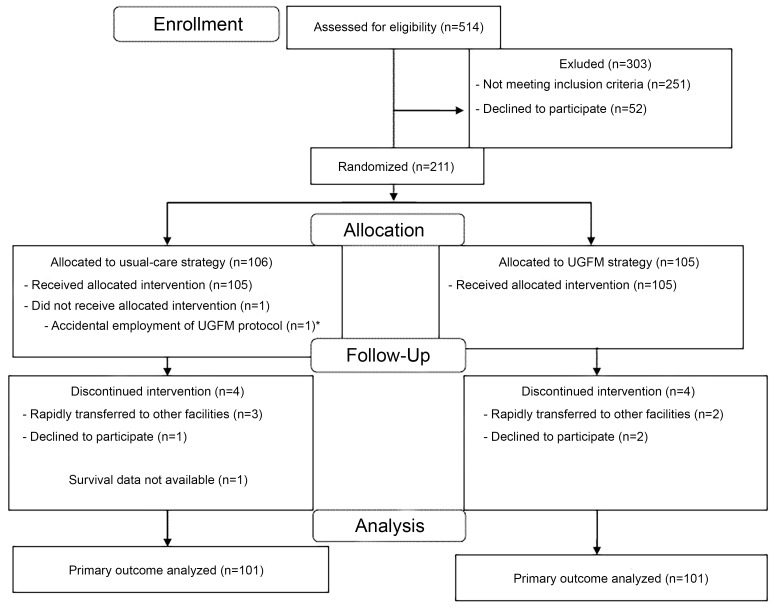
The CONSORT diagram demonstrating patient flow in both treatment groups. Reasons for patient exclusion are shown in Table S1 in the Supplemental material. *This patient was finally analyzed in the usual-care arm according to intention-to-treat analysis. *UGFM*, ultrasound-guided fluid management.

In accordance with the data safety monitoring board, the investigators decided to stop the trial before the target number of participants was recruited due to the possible ineffectiveness of the intervention and safety concerns. In total, 514 patients were screened for eligibility; 106 and 105 eligible patients were randomized to the usual-care and UGFM treatment arms, respectively. In summary, there were 101 patients in each treatment group available for intention-to-treat analysis. The patient flow chart and exclusion details are shown in Figure 1 and Table S1 of the Supplemental material. There was no significant difference in the demographic data of patients between the two treatment groups as demonstrated in Table 1 and Table S2 of the Supplemental material.

### Study outcome analysis

**Figure 2:**
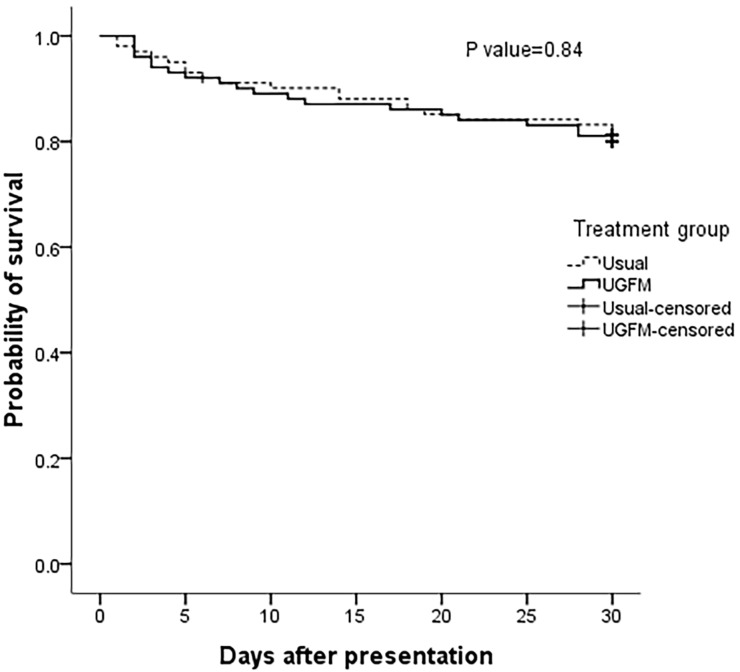
Kaplan–Meier curves showing the probability of survival among patients treated with ultrasound-guided fluid management or the usual-care strategy (intention-to-treat analysis). The hazard ratio was 0.94 (95% confidence interval [CI], 0.50 to 1.75), P>0.05 by log rank test.

There was no significant difference in 30-day mortality between the two groups (Figure 2, 18.8% and 19.8% in the usual-care and UGFM strategies, respectively; *p* > 0.05 by log rank test). For secondary outcome analyses, we did not find a significant difference between the treatment groups in six-hour lactate clearance, SOFA score at 72 hours or the length of hospital stay. However, the rate of vasopressor use and the cumulative fluid administration in 24 hours was lower in the UGFM arm. The comparisons of the study variables are shown in Table 2. Inferior vena cava targets were achieved at least two times in 68.3% of the patients in UGFM arm. The ultrasonographic IVC parameters of the patients in this group are summarized in Table S3 of the Supplemental material.

### Subgroup analysis

**Figure 3:**
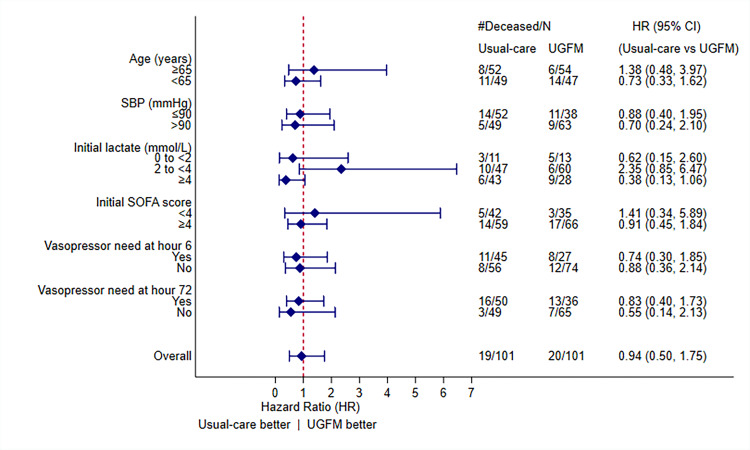
Forest plot for prespecified subgroup analyses of primary outcome adjusted for age, systolic blood pressure at presentation, initial lactate level, initial sequential organ failure assessment score, and vasopressor need at hour-6 and hour-72. All P-values for interaction are >0.05. *SBP*, systolic blood pressure; *SOFA*, sequential organ failure assessment; *HR*, hazard ratio; *CI*, confidence interval.

We performed prespecified analysis among different patient subgroups. However, we did not find a significant survival benefit in any specific subgroup. Whereas a positive survival trend in the UGFM treatment arm was discovered for patients with slight elevation of initial blood lactate (2 to <4 mmol/L), the analysis also revealed a potential negative effect of the intervention on those with initial lactate ≥4 mmol/L. However, neither of the subgroups reached statistical significance. A forest plot of the subgroup analysis and related information is shown in Figure 3.

## DISCUSSION

The use of point-of-care ultrasound (POCUS) is helpful in rapidly identifying causes of shock in the ED.^26^,^27^ Moreover, it is useful in determining fluid responsiveness during resuscitation in critically ill patients through the measurement of respiratory change in IVC diameter.^28^–^30^ Although some investigators discourage the routine use of IVC ultrasound to guide fluid therapy in critically ill patients,^31^ this study, to our knowledge, was the first to specifically investigate the effect of this intervention on patient survival. Ultimately, we did not find an improvement in the clinical outcome of septic patients treated with the UGFM strategy in our setting. Similarly, a recent study showed that the early use of POCUS protocol did not result in a survival benefit in patients with undifferentiated hypotension.^32^

The results from an impactful research on a large database of the ED patients who had been admitted to the ICUs with SS showed that the use of large fluid volume (over five liters) during the first day of SS resuscitation was associated with increased risk of hospital mortality.^33^ According to our results, the median volume of fluid administered to both groups of patients was still in a low range (less than 5000 mL in the first 24 hours) and the difference of the 24-hour fluid amount between the study arms, although statistically significant, was not substantial (median = 4800 vs 4080 mL in the usual-care and UGFM groups, respectively). Thus, the early-phase resuscitative fluid amount used in either group did not reach the harmful threshold that may have produced an increased mortality risk. This could possibly result in the similar mortality rates no matter which treatment strategy had been performed. However, our study revealed even less fluid amount given in the UGFM group compared to that of the usual-care treatment.

Our in-depth study analysis showed that, regardless of the treatment strategies, the median amount of fluid used during the initial phase of resuscitation in non-survivors was strikingly higher than that of survivors. (Table S4 of the Supplemental material). These secondary findings imply that more volume of initial resuscitative fluid was associated with mortality probability. Thus, physicians should be aware of unnecessary or irrational fluid bolus during their resuscitation practice. The use of respiratory change of IVC diameter measured by POCUS during the initial phase of sepsis resuscitation may generate the test characteristics that prospectively direct physicians not to “overload” patients with a cautious fluid restriction. Current trends of fluid resuscitation in septic shock advocates the minimization of fluid therapy and prevention of fluid overload.^34^–^36^ Furthermore, previous studies demonstrated that dynamic assessments to guide fluid administration can reduce the amount of fluid and potentially improve outcomes of patients with septic shock.^37^–^38^ Our study is likely to support these concepts.

Interestingly, we found a significantly lower rate of vasopressor use in patients treated with UGFM, while a recent study revealed a higher incidence of vasopressor need in septic patients resuscitated with a restrictive fluid strategy.^39^ Previous data have shown the detrimental effects of a large fluid bolus on physiologic changes and clinical outcomes of septic patients.^40^–^42^ An animal study demonstrated that sheep with endotoxemic shock and large-volume resuscitation needed a higher vasopressor dose to maintain their mean arterial pressure.^43^ Our results also revealed a possible association trend, although not statistically significant, toward the decrease in ventilator requirement in the UGFM group. However, there was conflicting data about the association between large amount of fluid volume and the requirement of MV in the resuscitation of SS patients.^44^, ^45^ The explanation for the reduction in vasopressor need and potential decrease in ventilator requirement during the treatment of septic patients in the UGFM group in our study remains unclear and needs further investigation.

The equivocal outcomes in the specific subgroups of SITH/SS patients also require additional scientific investigation in a larger population. According to our results in this ED, the initial use of respirophasic change in IVC diameter with POCUS in resuscitating SITH/SS did not improve the overall survival probability of patients compared to those treated with the usual-care strategy. It also did not improve lactate clearance or SOFA score. However, it was associated with a reduced amount of IV fluid given during the initial resuscitation.

## LIMITATIONS

Our study had noteworthy limitations. First, this study was conducted with the specific study protocol at a single, tertiary-care hospital. The results may not be generalizable to other settings with various resuscitative techniques or protocols or if different target parameters are set. Second, since our recruited patients had median initial SOFA scores of 4, and one-third of them required a vasopressor or MV during initial phase of their treatment, physicians should use cautious clinical judgment or different approaches when treating more severe septic shock patients in their practice. Third, bias may have occurred during patient allocation, data collection or outcome measurement due to the unconcealed nature of the study interventions, although this was minimized by the appropriate randomization. Moreover, the primary study outcome was the mortality rate of the patients, which is generally unaffected by blinding of the assessors.

Fourth, the higher rate of achieving the IVC collapsibility targets in patients treated in the ultrasound-guided arm may have affected the outcome. Finally, although the standard protocol and location of IVC diameter measurement was determined before the study, the interpersonal variation and sampling position may have affected the consistency of IVC diameter measurement.^46^, ^47^ However, the fair interrater reliability of IVC measurement was demonstrated among the emergency physicians,^48^, ^49^ and our study reflects real-life practice in dynamic EDs.

## CONCLUSION

In our ED setting, where a relatively restricted amount of IV fluid administration is generally practiced, we did not demonstrate the benefit of the use of respiratory change of IVC diameter determined by point-of-care ultrasound to guide initial fluid resuscitation in SITH/SS patients in the ED in improving the 30-day survival probability or other clinical parameters compared to the usual-care strategy. However, it was associated with less amount of fluid used. Further studies are required to identify the optimal physiologic targets and fluid resuscitation approach in the initial treatment of sepsis-induced hypoperfusion and septic shock patients in the ED.

## Supplementary Information



## Figures and Tables

**Table 1:** Demographic data of the patient cohort.

Clinical parameters and patient characteristics	Total n=202(100%)	Usual care n=101 (100%)	UGFM n=101 (100%)	*p*-value (95% CI)
At presentation				
Female gender	86(42.6)	38(37.6)	48(47.5)	0.14
Age, years (means±SD)	64.5±18.5	63.7±16.8	65.3±20.1	0.52
Body weight, kilograms (means±SD)	55.2±12.2	55.6±12.8	54.7±11.7	0.55
Triage-to-antibiotic time (minutes), median (IQR)^a^	59(41–76)	54.5(40–68.5)	60.5(44–84.5)	0.09
SBP at presentation, mmHg (means±SD)	102.4±28.9	99.2±26.9	105.7±30.4	0.11
≤ 90	90(44.6)	52(51.5)	38(37.6)	0.06
≤ 90 without hyperlactatemia (≥ 2 mmol/L)	23(11.4)	11(10.9)	12(11.9)	0.83
SOFA score (points), median (IQR)^a^	4(3,6)	4(3,6)	4(3,6)	0.82
≥ 2	181(89.6)	92(91.1)	89(88.1)	0.49
≥ 4	125(61.9)	59(58.4)	66(65.3)	0.31
Initial lactate (mmol/L)^a^	3.3(2.4–4.6)	3.6(2.4–5.6)	3.2(2.3–4.1)	0.08
≥ 2	178(88.1)	90(89.1)	88(87.1)	0.66
≥ 2 without SBP ≤ 90 mm Hg	111(55.0)	49(48.5)	62(61.4)	0.07

*UGFM*, ultrasound-guided fluid management; *CI*, confidence interval; *mmo/L*, millimoles per liter; *SD*, standard deviation; *IQR*, interquartile range; *SBP*, systolic blood pressure; *mm HG*, millimeters mercury.

* P-value < 0.05.

a Mann-Whitney U test.

**Table 2:** Results of the patient cohort and comparisons between treatment groups.

Parameters	Total n=202 (100%)	Usual-care n=101(100%)	UGFM n=101 (100%)	*p*-value (95%CI)
At 6 hours				
6-hour lactate (mmol/L), median (IQR)^a^†	1.9(1.1–3.2)	2.0(1.1–3.5)	1.8(1.1–2.8)	0.32
6-hour lactate clearance (%), median (IQR)^a^	37.8(10.3–60.0)	35.9(16.3–65.5)	39.2(7.4–60.0)	0.86
> 10%	150(75)	78(78)	72(72)	0.33
> 50%	75(75)	34(34)	41(41)	0.31
Normalization of 6-hour lactate	108(54)	51(51)	57(57)	0.40
Vasopressor use				
At 6 hours	72(35.6)	45(44.6)	27(26.7)	0.008*
Missing data	0	0	0	.
At 72 hours	86(43.0)	50(50.5)	36(35.6)	0.034*
Missing data	2(1)	2(2.0)	0(0)	.
Mechanical ventilator use				
At 6 hours	35(17.3)	22(21.8)	13(12.9)	0.094
Missing data	0	0	0	.
At 72 hours	53(26.5)	30(30.0)	23(23.3)	0.26
Missing data	2(1)	1(1.0)	1(1.0)	.
Renal replacement therapy at 72 hours	13(6.6)	5(5.1)	8(8.0)	0.41
sCr at 72 hours (mg/dL), median (IQR)^a^	0.8(0.6–1.1)	0.8(0.6–1.0)	0.8(0.6–1.1)	0.65
Acute kidney injury‡ (%)	6(3.6)	2(2.3)	4(5.0)	0.35
Missing data	35(17.3)	14(13.9)	21(20.8)	.
Cumulative fluid used (mL), median (IQR)^a^				
At 6 hours	2,400(1,839–2,950)	2,600(2,300–3,220)	1,900(1,500–2,570)	<0.001*
Amount of fluid per kilogram (mL/kg)	44(33–57)	48(38–63)	36(28–49)	<0.001*
Missing data	0	0	0	.
At 24 hours	4,507(3,508–5,716)	4,800(3,810–6,410)	4,080(2,990–5,255)	<0.001*
Amount of fluid per kilogram (mL/kg)	85(61–113)	88(67–123)	79(51–102)	0.005*
Missing data	24(11.9)	12(11.9)	12(11.9)	.
At 72 hours	7,530(5,500–10,266)	7,702(5,900–11,275)	7,300(5,040–9,200)	0.044*
Amount of fluid per kilogram (mL/kg)	149(100–199)	156(104–204)	142(98–193)	0.13
Missing data	30(14.9)	18(17.8)	22(21.8)	.
SOFA score at 72 hours (points), median (IQR)^a^	3(1–6)	3(1–7)	3(1.75–5)	0.91
Changes in SOFA score (points)	1(−1 to 3)	1(−1 to 2)	1(−1 to 3)	0.85
< 2 points (%)	47(28.7)	28(32.6)	19(24.4)	0.25
Missing data	38(18.8)	15(14.8)	23(22.8)	.
Length of stay (days), median (IQR)a	8(5–16)	8(5–16.5)	8(4–15)	0.39
30-day mortality (%)	39(19.3)	19(18.8)	20(19.8)	0.84§

Note: Changes in SOFA score, SOFA score at presentation minus SOFA score at 72 hours.

* P-value < 0.05.

a Mann-Whitney U tes.t

† Data of 6-hour lactate among 100 patients in usual-care group and 100 patients in UGFM group was available for further calculation and analyses.

‡ Defined by an absolute increase in serum creatinine (sCr) at 72 hours after presentation at least 0.3 mg/dL (sCr at 72 hours minus sCr at presentation ≥ 0.3).

§ Log rank test, the hazard ratio was 0.94 (95% confidence interval, 0.50 to 1.75).

*UGFM*, ultrasound-guided fluid management; *SD*, standard deviation; *IQR*, interquartile range; *mg/dL*; milligrams per deciliter; *mL/kg*, milliliters per kilogram; *SOFA*, sequential organ failure assessment.
