# Supplementary material for: Randomized Controlled Trial of Ultrasound-guided Fluid Resuscitation of Sepsis-Induced Hypoperfusion and Septic Shock
Source: West J Emerg Med. 2021 Feb 10;22(2):369–78. doi: 10.5811/westjem.2020.11.48571 (PMC7972359; doi:10.5811/westjem.2020.11.48571)
Supplement: Supplementary file 1 [file wjem-22-369-s001.doc]

**Table S1. Reasons for patient exclusion.**

| Reasons for exclusion | Number of patients (cases) |
| --- | --- |
| Congestive pulmonary edema | 66 |
| Poor systolic cardiac function | 18 |
| Right-side heart diseases | 11 |
| Intra-abdominal pathologies/failure to identify the IVCs | 8 |
| Significant obesity | 12 |
| Severe airway diseases | 6 |
| End-stage renal diseases | 34 |
| Referred from other healthcare facilities | 12 |
| Active hemorrhage (e.g. gastrointestinal bleeding, hemoperitoneum) | 28 |
| Duplicated cases | 14 |
| Do-not-resuscitate living will or terminal illnesses | 42 |
| Total | 251 |


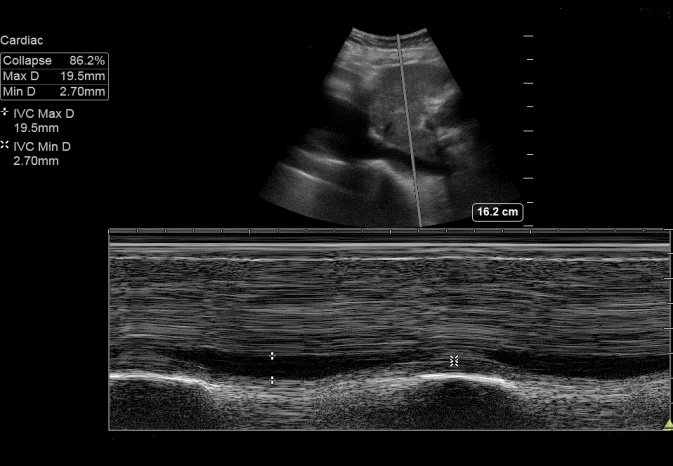

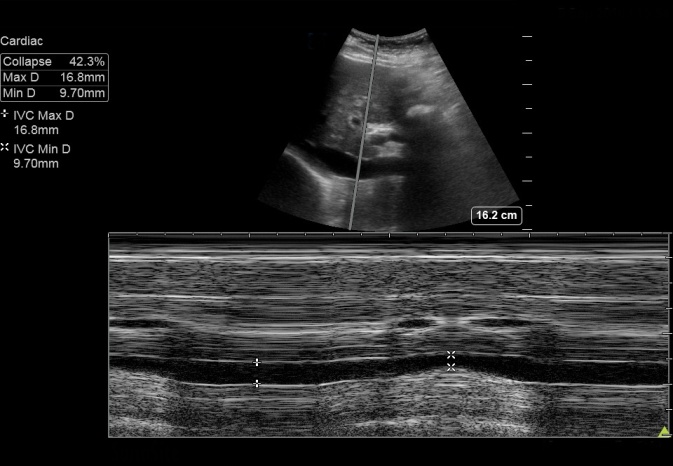


**Figure S1A Figure S1B**

**Figure S1.** The M-mode ultrasonography of an IVC at about 2 centrimeters distal to the confluence of hepatic vein in a spontaneously-breathing septic shock patient demonstrating a markedly collapsed IVC with IVCCI = [(19.5-2.7)/19.5]×100% = 86.2% **(Figure S1A)**. When a bolus of 1,500 ml (30 ml/kg) normal saline was given to this patient, IVCCI was decreased to 42.3% **(Figure S1B).**

**Table S2.** Supplemental demographic data of the patient cohort.

| Clinical parameters and patient characteristics | Total  N=202(100%) | Usual care  N=101 (100%) | UGFM  N=101 (100%) | p value  (95%CI) |
| --- | --- | --- | --- | --- |
| Site of infections  - Pneumonias  - Urinary tract  - Hepatobiliary and gastrointestinal  - Primary bacteremia  - Skin and soft tissue  - Systemic infections  - Others | 56(27.7)  47(23.3)  41(20.3)  39(19.3)  13(6.4)  3(1.5)  3(1.5) | 28(27.7)  22(21.8)  20(19.8)  19(18.8)  9(8.9)  2(2.0)  1(1.0) | 28(27.7)  25(24.8)  21(20.8)  20(19.8)  4(4.0)  1(1.0)  2(2.0) | .  .  .  .  .  .  . |
| Underlying conditions   - Diabetes mellitus - Hypertension - Cerebrovascular diseases - Malignancy - Receiving Chemotherapy - Neutropenia - Immunosuppressive drug use - Others - More than one disease | 64(31.7)  80(39.6)  52(25.7)  70(34.7)  34(16.8)  19(9.4)  25(12.4)  64(31.7)  128(63.4) | 35(34.7)  41(40.6)  24(23.8)  41(40.6)  18(17.8)  8(7.9)  15(14.9)  26(25.7)  63(62.4) | 29(28.7)  39(38.6)  28(27.7)  29(28.7)  16(15.8)  11(10.9)  10(9.9)  38(37.6)  65(64.4) | 0.36  0.77  0.52  0.08  0.71  0.47  0.29  0.07  0.77 |
| Previous hospitalization within 90 days | 81(40.1) | 39(38.6) | 42(41.6) | 0.67 |
| Antibiotic treatment within 30 days | 64(31.7) | 30(29.7) | 34(33.7) | 0.55 |
| CBC at presentation  - WBC count (×1000 per mm^3^)  - Hemoglobin (g/dl)  - Platelet (×1000 per mm^3^) | 12.6±9.0  10.9±2.6  233±141 | 13.8±10.3  11.0±2.8  230±147 | 11.9±7.3  10.7±2.3  236±135 | 0.12  0.49  0.77 |
| Blood chemistry  - Serum creatinine (mg/dl)^b^  - Total bilirubin (mg/dl)^b^  - Serum sodium (meq/L)  - Serum potassium (meq/L)  - Serum bicarbonate (meq/L) | 1.1(0.8,1.5)  0.9(0.5,1.7)  134.7±9.6  3.8±0.7  18.9±4.4 | 1.1(0.8,1.5)  0.8(0.5-1.4)  134.5±10.8  3.8±0.7  18.4±4.8 | 1.1(0.8,1.4)  0.9(0.6-2.1)  135.0±8.3  3.8±0.7  19.4±3.9 | 0.39  0.19  0.71  0.83  0.12 |
| Blood culture growth  - Gram positive  - Gram negative | 54(26.7)  15(7.4)  39(19.3) | 26(25.7)  6(5.9)  20(19.8) | 28(27.7)  9(8.9)  19(18.8) | 0.75  .  . |
| Adjunctive treatment  - Central venous catheterization  Missing data  - Packed red cell transfusion  Missing data | 32(16.9)  13(6.4)  58(30.7)  13(6.4) | 19(19.8)  5(5.0)  30(31.3)  5(5.0) | 13(14.0)  8(7.9)  28(30.1)  8(7.9) | 0.29  .  0.87  . |

**Table S3.** Ultrasonographic IVC parameters of the patients in UGFM group (N=101).

| Parameters  Time of  assessment | Minimal diameter (mm., mean±SD) | Maximal diameter (mm., mean±SD) | IVCCI (%, mean±SD) | Proportion of the patients who achieved the IVC targets* (%) | Missing data N(%) |
| --- | --- | --- | --- | --- | --- |
| - At presentation  - At 2 hours  - At 4 hours  - At 6 hours | 6.9±5.3  10.2±5.4  10.9±5.3  11.0±5.3 | 12.7±5.2  15.0±5.0  15.1±5.0  15.2±4.9 | 50.7±27.5  35.0±22.8  30.2±20.0  30.4±20.8 | 20.3  64.4  72.1  67.5 | 0  0  15(14.9)  18(17.8) |

**Notes**: Among 101 patients in this group, sixty-nine patients (68.3%) achieved the IVC targets at least two times of the assessment. 12 patients (11.9%) never achieved the IVC goals during all individual assessment. Four patients were intubated with subsequent deep sedation (one patient at hour-0, one patient at hour-2 and two patients at hour-4), IVC distensibility index was used accordingly.

*IVCCI ≤ 40% or IVCDI ≤ 18%

mm.=millimeters, SD=Standard deviation, IVC=inferior vena cava, IVCCI=IVC collapsibility index, IVCDI= IVC distensibility index.

**Table S4.** Cumulative fluid amount at 6, 24, and 72 hours after presentation among survivors and non-survivors.

| Parameters | Non-survivors  n=39 | Survivors  n=163 | p value  (95%CI) |
| --- | --- | --- | --- |
| - Cumulative fluid used (ml), median (IQR)^a^  - At 6 hours  Amount of fluid per kilogram (ml/kg)  Missing data  - At 24 hours  Amount of fluid per kilogram (ml/kg)  Missing data  - At 72 hours  Amount of fluid per kilogram (ml/kg)  Missing data | 2500(2200,3100)  52(43,67)  0  5370(4469,6566)  106(87,140)  3(7.7)  10573(8063,12691)  198(154,296)  9(23.1) | 2300(1700-2800)  41(31,55)  0  4217(3200,5481)  79(57,103)  21(12.9)  7030(5253,9200)  134(92,179)  31(19.0) | 0.028*  <0.01*  .  <0.001*  <0.001*  .  <0.001*  <0.001*  . |
| Patients with 24-hour fluid > 5000 ml, n(%)  Missing data | 21(58.3)  3(7.7) | 44(31.0)  21(12.9) | 0.002[^§^](https://en.wikipedia.org/wiki/Section_sign)  . |

**Notes**: IQR=Interquartile range

* p value < 0.05

^a^ Mann-Whitney U test

[§](https://en.wikipedia.org/wiki/Section_sign) Chi-squared test, the odd ratio is 3.1 (95% confidence interval [CI], 1.5 to 6.6).
